# Power optimization of a photovoltaic system with artificial intelligence algorithms over two seasons in tropical area

**DOI:** 10.1016/j.mex.2022.101959

**Published:** 2022-12-10

**Authors:** Amadou BA, Alphousseyni NDIAYE, El hadji Mbaye NDIAYE, Senghane MBODJI

**Affiliations:** Department of physics, University Alioune Diop, Bambey, Senegal

**Keywords:** Artificial Neural Network, Adaptive Neuro-Fuzzy Inference System, Photovoltaic, Maximum Power Point Tracking, ANN, Artificial Neural Network, ANFIS, Adaptive Neuro-Fuzzy Interence System, PV, Photovoltaic, MPPT, Maximum Power Point Tracking, Power Optimization of a Photovoltaic System with Artificial Intelligence Algorithms over two seasons in tropical area

## Abstract

Power output from the PV module changes continuously with time depending upon the climatic condition. This changes are most important in tropical area like Senegal due to the variation of the seasons (dry and rainy). Furthermore, different types of maximum power point tracking (MPPT) algorithm are presented in literature in order to get maximum output from the PV system. They can be summarized in two categories: classical and intelligent methods. The classical methods in no uniform weather condition are not efficient and an important loss of energy is showed. However, faced to this problematics like energy loss and no uniform weather conditions an Adaptative methods is used to optimize the PVs energy. In this study, two intelligent controllers based on artificial neural networks (ANN) and Adaptive Neuro Fuzzy Inference System (ANFIS) are proposed to optimize the PVs production in non-uniform weather conditions and compared in order to show the most powerful model. For the ANN, the main challenge is to find the optimal neural in the hidden layer and in the paper, it is obtained using evaluator factor like mean squared error (MSE). These techniques using artificial intelligence (AI) algorithms are used for power optimization of a photovoltaic system are trained and validated with real data from a photovoltaic micro power plant in dry and rainy season installed at polytechnic high school of Dakar. The performances of the controllers to optimize the PVs power are evaluated during the dry and rainy seasons. Simulation results show that the ANFIS MPPT controller is more efficient and robust than ANN in non-uniform weather conditions.

They have the ability of generalization and adaption to each meteorological conditions. These bullet summarize the applied methodology

• A real electrical characteristics of photovoltaic panel are used for learning and validation of the controllers.
• A comparative study of the methods in two different season is done.
• ANFIS gives best performance in weather conditions compared to the ANN.

## Highlights

• A real electrical characteristics of photovoltaic panel are used for learning and validation of the controllers.
• A comparative study of the methods in two different season is done.
• ANFIS gives best performance in weather conditions compared to the ANN.

## Graphical Abstract

Graphical Abstract.

**Figure:**
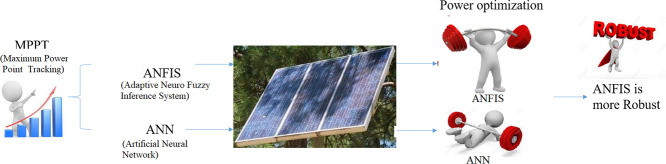

Specifications table

**Table:** 

Subject Area:	Energy
More specific subject area:	- Solar Energy - MPPT controller based on intelligent controllers to optimize photovoltaic power
Method name:	*Power Optimization of a Photovoltaic System with Artificial Intelligence Algorithms over two seasons in tropical area*
Name and reference of original method:	*http://dx.doi.org/10.1016/j.rser.2016.11.125* *DOI:**10.30521/jes.434224*
Resource availability:	*N/A*

## Introduction

The demand on power production has well increased in recent years. On the other hand, power plants, reliant on fossil fuel reserves, are not enough to meet the electricity demand because of the exhaustion of these reserves. The urgency is to call on to another energy resource like renewable energy. The requirement of PV based generation is getting increased in both standalone application and in grid connected system. In general, the two main problems with PV power generation systems are the low conversion efficiency around 10-20 % [1] and that electrical power generated by a PV panel varies with weather conditions. The PV system gives its maximum power at a particular point namely Maximum PowerPoint (MPP).

In the literature, various control techniques commonly known as Maximum Power Point Tracking (MPPT) controls [2,3] have been developed to track the MPP and optimize the efficiency of photovoltaic system (PV). A classification of the most applied MPPT algorithms is done in [4] and based on different norms like tracking techniques, sensing implementation, and modernity. The perturb and observe (P&0), incremental conductance (INC), and hill climbing (HC) algorithms are massively used to optimize PVs power. This techniques have various drawbacks, like slow tracking, steady-state oscillations at maximum power points and low efficiency. Many methods based on artificial intelligence have been developed to improve the conversion efficiency of PV systems to overcome those disadvantages. These techniques are fuzzy logic (FL), genetic algorithms (GA), artificial neural networks (ANN), the Optimization swarms of particles (PSO) [5] and the Adaptive Neuro Fuzzy Inference System (ANFIS), which is a hybrid neuro-fuzzy controller. In research work done by Meddour et al. [6] a comparative study of the PSO and P&O methods to extract the maximum power of a PV panel is made. The objective is to overcome the disadvantages of the P&O technique as the convergence speed and the oscillations around the Maximum Power Point (MPP) in no-uniform weather conditions. Simulation results show good performance of the meta-heuristic PSO method compared to the conventional P&O.

A MPPT control based on FL is carried out by Farajdadian et al. [7] to optimize the yield of a photovoltaic system. The main drawback of this method is the choice of the membership function where four optimization algorithms are used to optimize the fuzzy membership (MF) and generate an appropriate duty ratio for the MPPT controller. Finally, to validate the performance of the optimized FL, it is compared with other techniques such as symmetric fuzzy logic controller (SLFC) and P&O methods. Simulations results showed a fast convergence speed and the decrease the oscillations in non-uniform weather conditions. The main advantage of the fuzzy-logic is the ability to adapt for the nonlinear conditions.

Nowadays ANN is massively used in many applications in PV system because of non-linearity in meteorological data. It has been used in prediction applications, in the forecasting or optimization of PVs power. ANN is more suitable compared with the statistical methods when a non-linear and complicated bonding exists between the data without any prior assumption. A MPPT method [8] based on ANN was also proposed for continuing the overall of the MPP in various weather conditions for a standalone PV system. The proposed method includes the PV characteristics scanning procedure with the ANN controller in the shading effect. The simulation results show that the proposed method is able to find the MPP and ensures rapid convergence towards this point with good efficiency estimated to 99.4% compared to the results obtained with P&0 command. A hybrid MPPT approach based on ANN-INC and ANN-P&O algorithm is also proposed [9]. Compared to conventional methods, it is noted a higher output power and less oscillations around the MPP with the RNA-INC controller. A new MPPT technique for partial shading condition (PSC) is introduced to optimize the efficiency of PV systems [10]. The proposed method based on ANN to predict the area of the global maximum power point (GMPP), and the classic P&O algorithm to track the exact position of the GMPP. Simulations results showed that the proposed method is more efficient than the P&O and FL MPPT considered, based on its ability to track the GMPP quickly and accurately. ANN give a good performance in optimization of PVs power but there main problematic is to find the appropriate configuration such as the number of neuron in the hidden layer.

A combination of the advantages of the (FL and ANN) can provide a better performance for the optimization methods. ANFIS is a hybrid neuro-fuzzy controller witch associate the (FL and ANN). In [11] the authors show the good performance of ANFIS control compared to a heuristic INC command to optimize the conversion efficiency of a PV system. The results show that the intelligent control displays a response time an order of 2.4 s and leads to an efficiency of 99.94 % compared to 94.41 % of the one obtained with INC control. The ANN has been widely used to model the complex relationships between inputs and outputs of nonlinear systems and simultaneously contributes to the optimization of the efficiency of photovoltaic systems. In [12] authors describe the use of an ANFIS to improve solar power of grid connected system.

A fractional order proportional integral derivative (FOPID) is used to generate the duty cycle through calculating the error within the reference voltage, the output of the ANFIS model and PV voltage. In terms of efficiency simulations results showed that, the ANFIS based FOPID controller is superior to the INC and ANFIS based PID. A comparative study between INC, PSO and ANFIS MPPT controllers to optimize the power of PV system is done in [13]. Simulation results have shown that ANFIS and PSO compared to the classical method. In this study, the authors have not done the evaluation in no-uniform weather condition such as the rainy season impact the MPPT methods. ANFIS gives best performance in term of response time and accuracy. A comparative study of ANFIS and ANN is done by Rim et al [14] for forecasting the PV power for various variation of the weather conditions. The simulation results showed satisfactory results were obtained using the ANN and the ANFIS models. But in this study, the authors don't study the ANN configuration like the choose of the number of neuron in the hidden layer. In case of our study we will use this two techniques for the optimization of PV autonomous system for two seasons in a tropical area.

Many researchers are done to optimize the power of the PV system to overcome the problematic of low conversion efficiency due to the intermittent of the PV source. ANN and ANFIS show there performance in weather conditions compared to the others MPPT techniques and are very powerful and robust because of their ability to adapt to nonlinear systems.

The main objective of this paper is to make a comparative study between ANN and ANFIS MPPT controller to optimize the power of a PV system in non-uniform weather conditions in tropical area.

This research was motivated in the first hand, by in non-uniform weather conditions few articles are published compared to the area of PSC [15] and no one in tropical area. And in the other hand, many researchers are done for optimization of the PVs power and this study will confirm the most powerful technique between ANN and ANFIS in tropical area.

A following contribution are reporting in this study:

• the database for the training and validation of these controllers is obtained by an experimental set up installed in Dakar (Senegal);
• the number of neurons in the hidden layer and training approach are found by optimization based on the training error (MSE).
• a daily weather conditions of two months Mars and August corresponding to dry and rainy seasons are used to evaluate the performances of the methods;
• this study show that ANFIS is most powerful of ANN in no uniform weather condition.

This paper is organized as follows. The methodology is study in Section 2. In Section 3 the simulation results are presented and the discussions are made. Finally, we concluded and the paper in Section 6 and given an outlook.

## Methodology

### Artificial neuron network (ANN)

ANN are massively used in nonlinear problems such as the PV systems. They have the capacity to learn from examples and deal with incomplete data. Once trained, ANN can perform fast prediction [16,17], optimization [18,19] and modelization of a system [20,21]. The use of ANN avoids solving complicated mathematical models of the systems moreover, it is not required to know the input/output relationships.

However, the non-linear P-V or I-V characteristics and the main variables that drive the solar PV are solar radiation and temperature. In this study an ANN is used to optimize the power of a photovoltaic system. A multilayer perceptron (MLP) is used that better fit with the non-linear systems [22]. In MLP, neurons are connected in multiple layers and the signal goes from the input to the output layers and between neurons of the same layer, there is no interconnection. The ANN structure is divided into three layers: an input layer gives by the number of input, an intermediate layer called hidden layer and an output layer gives by the number of output. More detail for the theory of modeling an ANN is done and can be found in other studies [23,24].

The main problematic of the ANN is to found the number of neuron in the hidden layer and the optimum approach for the training. Several methods have been used to optimize the number of neuron in the hidden layer [25]. It should be noted that this structure is chosen after several tests in order to increase the accuracy of the obtained neural network. We begging by fixing the number of approach and we change the number of the neuron in the hidden layer on the one hand Table 1 and fixing the number of the optimum neuron and we vary the number of apoach on the other hand Table 2. The performance of the network can be evaluated using Mean Squared Error (MSE) as Eq. (1):

(1) $$MSE=\frac{1}{n}\sum_{i=1}^{n}{\left(P_{opt}-P_{pred}\right)}^{2}$$

**Table 1:** Choose of the number of hidden neuron.

Number of approach	Number of neuron	MSE
1000	5	$9.083\;10^{-6}$
1000	10	$8.81\;10^{-6}$
1000	15	$9.35\;10^{-6}$
1000	20	$9.87\;10^{-6}$

**Table 2:** Choose of the number of approach.

Number of approach	Number of neuron	MSE
100	10	$9.25\;10^{-6}$
200	10	$8.87\;10^{-6}$
300	10	$9.16\;10^{-6}$
400	10	$9.45\;10^{-6}$

Where $P_{opt}$ is the desired power of the PV and $P_{pred}$ is the predicted power by the ANN and n the size of the database.

The best MSE during the training process is equal to $8.81\;10^{-6}$ and $8.87\;10^{-6}$ respectively for 10 neuron and 200 approach Table 1 and 2. Finally the optimum number of neurons in hidden layer is 10 neurons because a small optimum performance is obtained. The structure of the ANN MPPT has two neurons for the input layer, teen neurons for the hidden layer and one neuron for the output layer. The inputs is the current and voltage of the PV and the output is the predicted power.

**Fig. 1:**
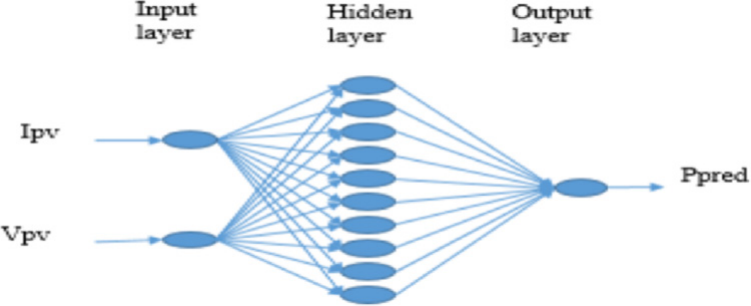
ANN MPPT configuration.

The proposed ANN-based MPPT controller consists of a three-layer feedforward network as shown in Fig. 1. The neural network is defined according to its structure which includes number of layers, numbers of neurons in each layer, type of activation function in each layer and interconnection between layers. There are several activation functions [26] and in the case of our ANN model the sigmoid function is used for the hidden layer. The particularity of this function is its first derivative value is positive and appropriate for the learning algorithm and its output is bounded between 0 and 1. A sigmoid function is defined by in the Eq. 2:

(2) $$S\left(x\right)=\frac{1}{1+e^{-x}}$$

### Adaptive neuro-fuzzy inference system (ANFIS)

**Fig. 2:**
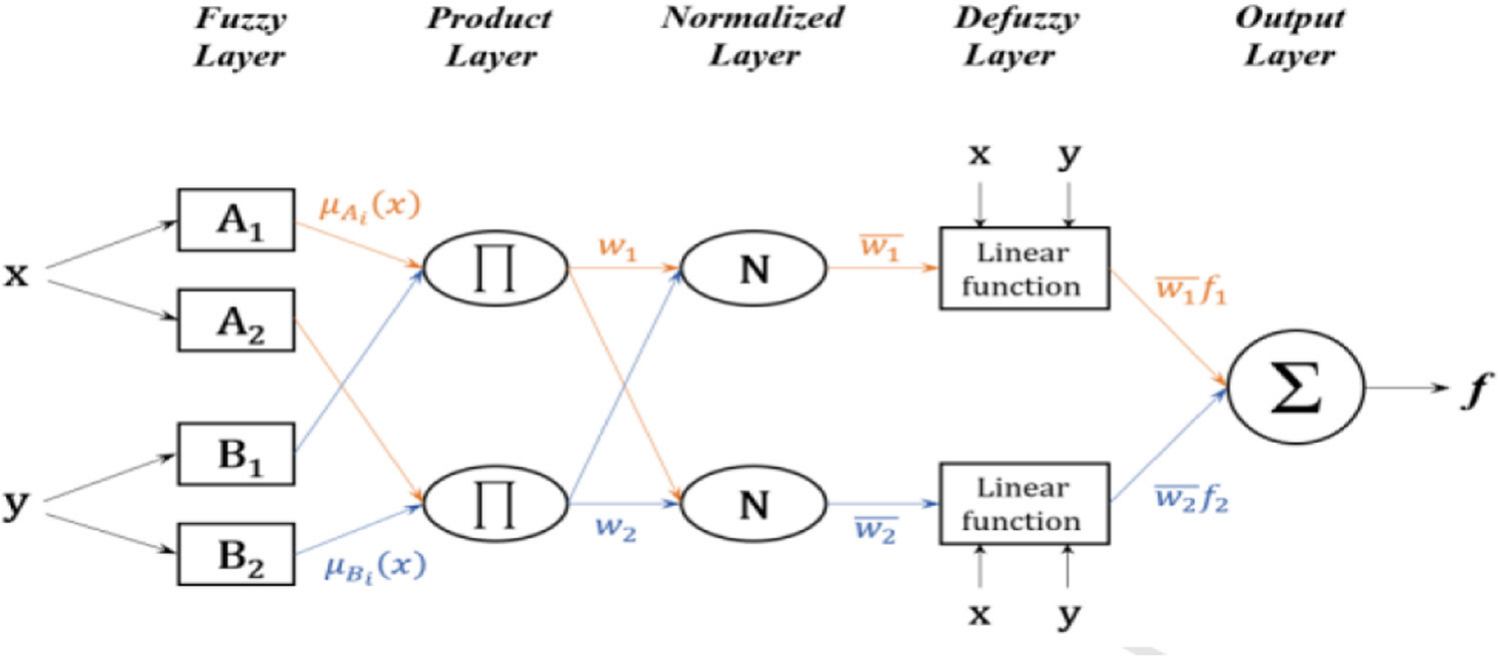
Structure of an ANFIS model.

Inspired by human brained functioning, the ANN from a database has the ability to learn and improve the performance a system. Compared to the ANN, Fuzzy logic FL has the capacity to understand and interpret a variables and is based on linguistic rule. The main motivation of using the ANFIS is to develop a powerful combination of the advantages of ANN and fuzzy systems. It is generally used in many applications such as the ANN in optimization and forecasting and controlling [27] a PV system. ANFIS implements a Takagi Sugeno FIS and has a five layered architecture as shown in Fig. 2. The first hidden layer is for fuzzification of the input variables and in the second hidden layer, it receives the outputs of fuzzification neurons and calculates its activation to compute the rule antecedent part. The third hidden layer normalizes the rule strengths followed by the fourth hidden layer where the consequent parameters of the rule are determined. Output layer computes the overall input as the summation of all incoming signals. ANFIS uses back-propagation learning to determine premise parameters (to learn the parameters related to membership functions) and least mean square estimation to determine the optimal parameters [12].

It structure composed of five layers, is described by the following equations. Each one models a layer. The Gaussian membership function is used in this work.

Layer 1:

(3) $$O_{i}^{k}=\mu A_{i}\left(I_{pv}\right)$$

(4) $$O_{i}^{k}=\mu B_{i}\left(V_{pv}\right)$$

Where:

(5) $$\mu A_{i}\left(I_{pv},\sigma \right)=\exp{\left[\;\frac{I_{pv}-\bar{I_{pv}}}{\sigma I_{pv}}\;\right]}^{2}$$

(6) $$\mu B_{i}\left(V_{pv},\sigma \right)=\exp{\left[\;\frac{V_{pv}-\bar{V_{pv}}}{\sigma V_{pv}}\;\right]}^{2}$$

Layer 2:

(7) $$W_{i}=\;\mu A_{i}\left(I_{pv},\sigma \right)\;\mu B_{i}\left(V_{pv},\sigma \right)$$

Layer 3:

(8) $$\bar{\omega_{i}}=\frac{W_{i}}{W_{1}+W_{2}}$$

Layer 4:

(9) $$O_{i}^{k}=\bar{\omega_{i}}\left(A_{i}x+B_{i}y+C_{i}\right)$$

Layer 5:

(10) $$O_{i}^{5}=\sum_{i}\bar{\omega_{i}}\;f_{i}$$

**Fig. 3:**
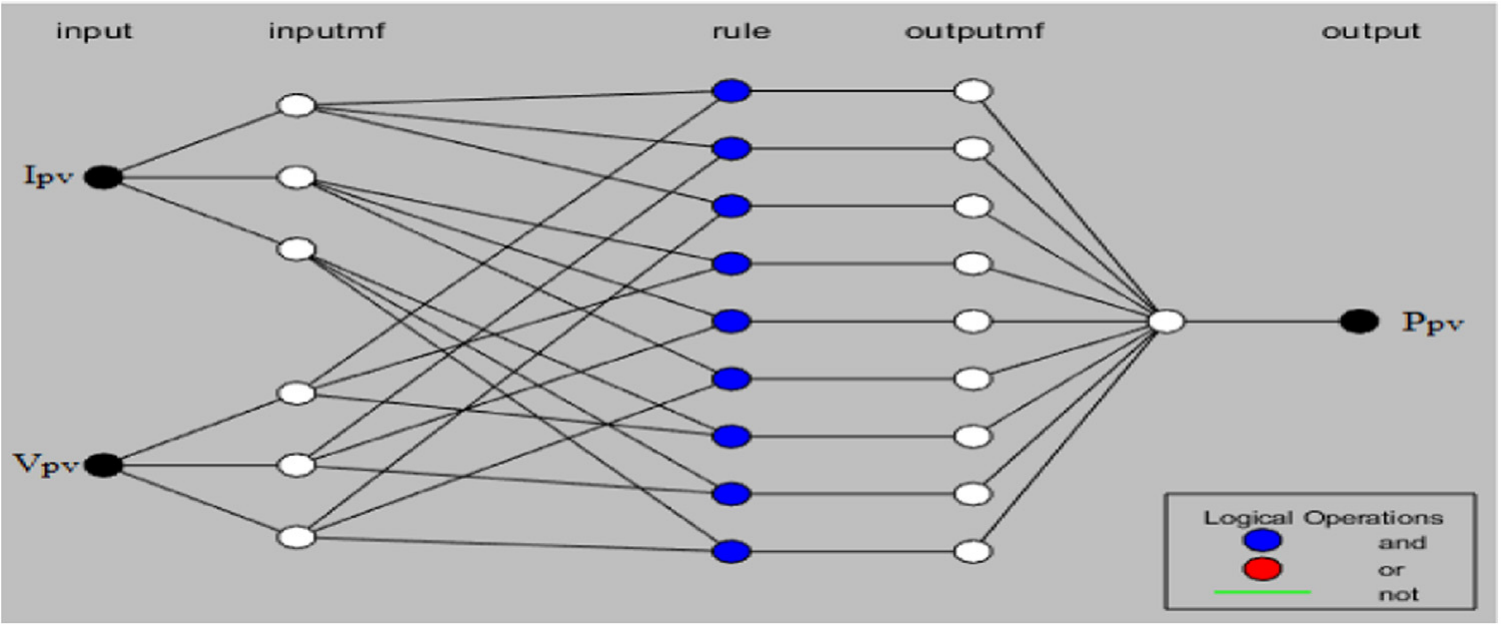
ANFIS structure generate by matlab.

Where: $A_{i}$, $B_{i}$, and $C_{i}$, are linguistic terms. σ is the standard deviation. As shown in Fig. 2, the framework of an ANFIS model contains a layers that are connected by fuzzy rules in the form of networks. Synergistic advantages can be achieved in one hybrid model, since ANFIS integrates the learning capacities of the ANN and reasoning abilities of the fuzzy system [28]. More details of the ANFIS can be found in other literature [11,13] such us the number of layer and rules, type of membership functions and learning algorithm This ANFIS model is obtained by Matlab software and those parameters are choose by an optimization techniques based on the tolerance error fixed at $10^{-4}$. The number of Membership function (MsF) is fixed at three (3) and generate nine (9) rules. The MsF for the input and the output layer is respectively Gaussian and Linear because give good performance such as low tolerance error compared to the others type and this comparative study is done in [29]. The structure proposed in this study consists of two inputs PV current and voltage, at the output, the controller will provide us the optimal PV power Fig. 3.

### Data collection

ANN and ANFIS are an approach based on artificial intelligence, our principle is based on learning and validation from a database. Nowadays several methods are available to generate data and computer science is an important step for the development of artificial intelligence. Among the data sources we can cite:

Applications: generate a data from a software, a rapports etc...

Open Data: correspond to the free provision of civil society data.

Open API (Application Programming Interface): are technologies for accessing data on the Internet. They make it possible to retrieve, for example, data made available by Google, Twitter, etc.

**Fig. 4:**
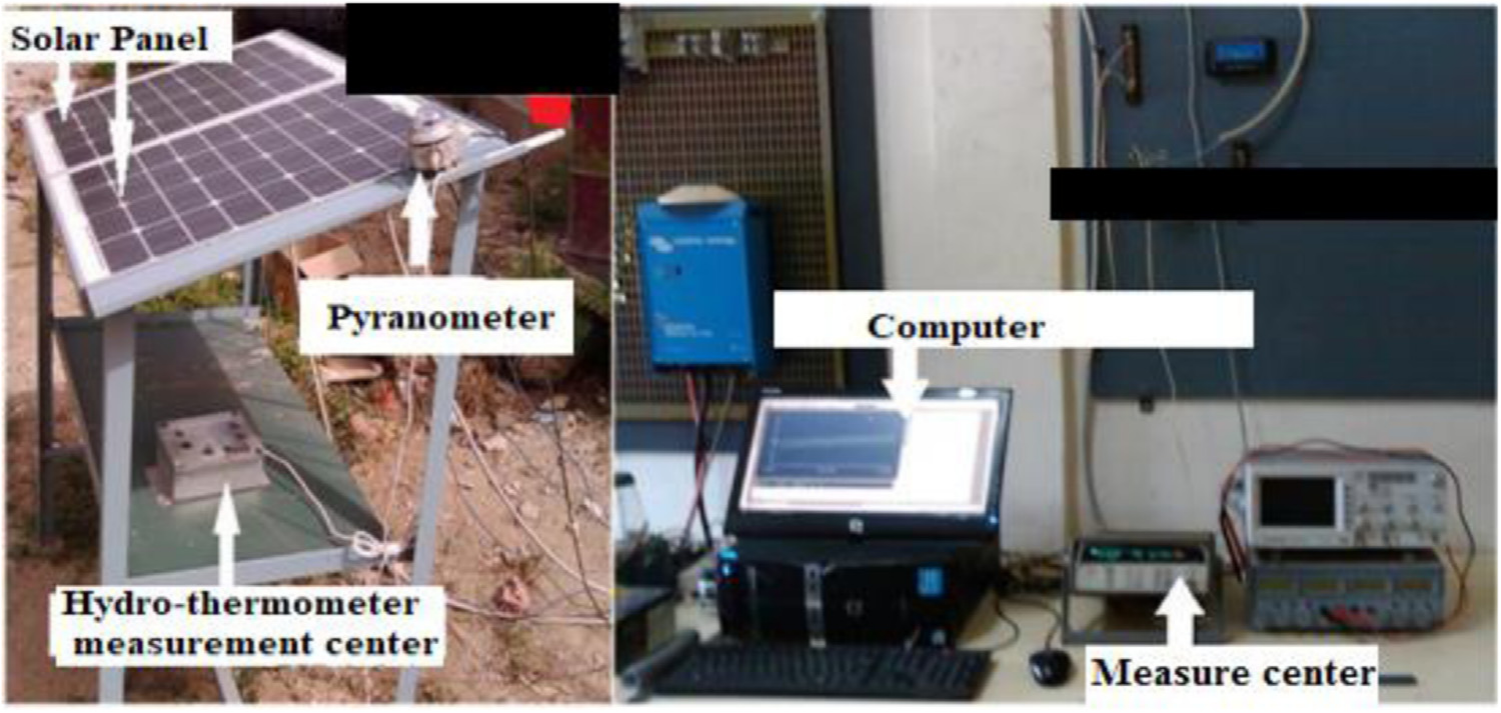
Experimental set up for database collection.

Web: is also a direct source of data. For this, you need a minimum knowledge in programming expertise to be able to do this known as web scraping, which consists of recovering data directly from the pages of the websites. In the case of our study the data required to train our models is obtained about an experimental set up Fig. 4. The components of are two panels whose we will measured the electrical characteristics with a measurement center, a pyranometer and a thermocouple are used to measure the irradiation of the site and the panels temperature and all the data are saved in a computer.

**Fig. 5:**
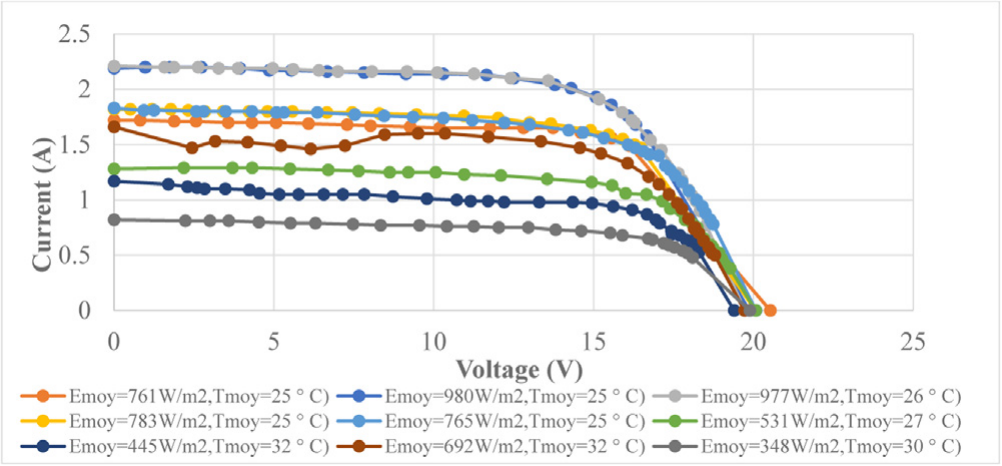
I-V curves of the PVs in different weather conditions.

**Fig. 6:**
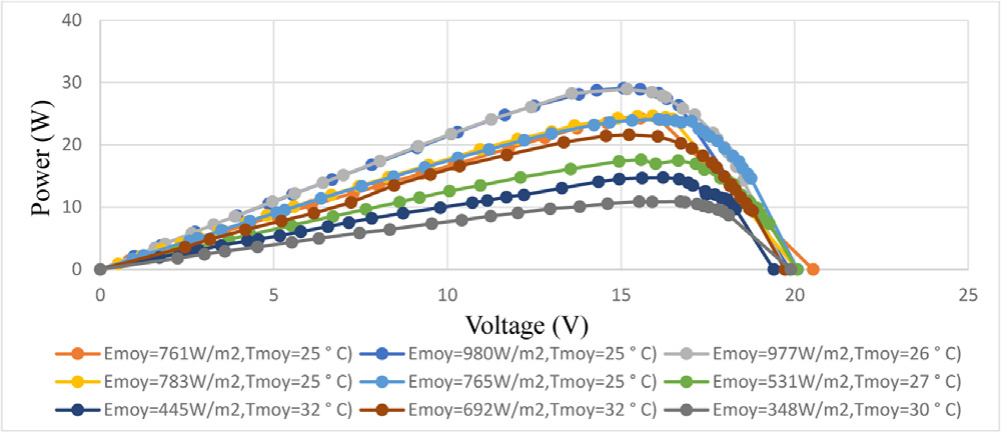
P-V curves of the PVs in different weather conditions.

This experimental set up is installed at Polytechnic Higher School of Cheikh Anta Diop University in Dakar, Senegal. Dakar is marked by two seasons, the rainy season which goes from July to October and the rest of the year is characterized by the dry season from November to June [30]. The data obtained are the characteristics of the panel in different weather conditions. Each radiation value (Em) is related to a temperature value (Tm). P-V and I-V characteristics of the solar panel in different profile of sunshine and temperature are shown in these Figs. 5 and 6.

**Fig. 7:**
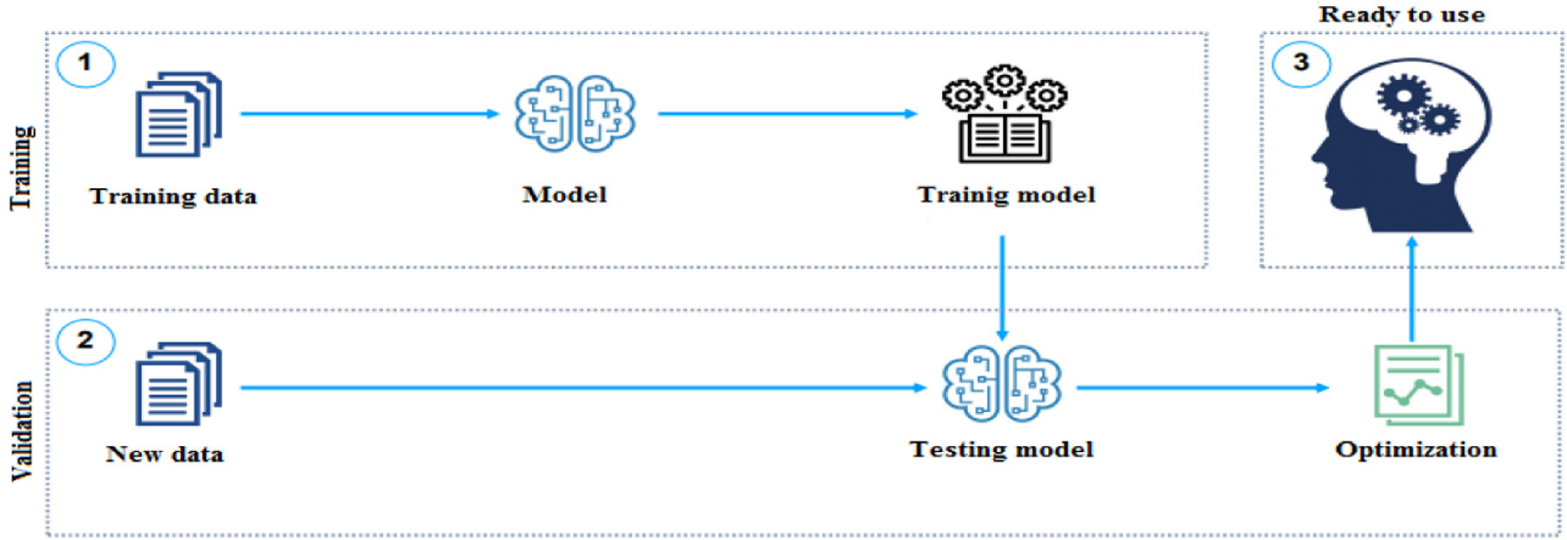
Training and validation processes of the model.

These figures (Fig. 5 and 6) show that the power of the photovoltaic is sensitive to the variations of the meteorological conditions like the radiation (Em) and the temperature (Tm) measured on the day of 18 March 2012. The panel gives it maximum power only for particular values of current and voltage that depend on weather conditions. During irradiance and temperature changing conditions the generating power also varies. To maintain the maximum power from the PV system, the MPPT algorithm is necessary to track the optimal point. To overcome this drawback of the classical MPPT methods, a two artificial intelligences MPPT controller will be developed to optimize the power of the panel. This data will be used to train the intelligent controller and for the validation the meteorological conditions measured in the August corresponding to the rainy season. After the data collection we are going to design the artificial intelligence controller and this step is summarized in the Fig. 7.

In the first step a pair (inputs, outputs) of data called learning examples, is presented to the model. This inputs are the current and the voltage of the panel and the output is the power obtain with the experimental set up. This step is repeated in several times until the model is able to predict the corresponding output. The second step is to present to the training model step first a new pair (inputs, output) and to analyze the performance of the model to predict the desired output and this step is namely the validation process. And the third step is the used of the training and validation model to optimize the PV panel power.

## Results and discussion

This study aims to predict the power of a solar panel at the Polytechnic higher school in Dakar using an artificial neural network. Two intelligence optimization techniques is used and a comparative study is done. To evaluate the performance of our controller we made a comparative study with two MPPT (ANN and ANFIS). To intuitively measure the performance of the proposed models and facilitate comparison of the approaches we compute, the mean absolute error (MAE), the mean-absolute percentage error (MAPE), and the root mean square error (RMSE) were introduced as follows whose expressions are presented respectively in Eqs. 11,12 and 13 [31]:

(11) $$MAE=\frac{1}{n}\sum_{i=1}^{n}\left|P_{opt}-P_{pred}\right|$$

(12) $$MAPE=100\frac{1}{n}\sum_{i=1}^{n}\left|\frac{P_{opt}-P_{pred}}{P_{opt}}\right|$$

(13) $$RMSE=\sqrt{\frac{1}{n}\sum_{i=1}^{n}{\left(P_{opt}-P_{pred}\right)}^{2}}$$

RMSE measures the average value of the errors, ranging from 0 to infinity, with lower values being better. The mean absolute percentage error MAPE is used for evaluating the optimization accuracy. It indicates how much percent the optimized value deviates from the actual one. The performance of any MPPT depends on its behavior under weather conditions such irradiation and temperature. The technical specifications of the PV panel are listed in Table 3 and the simulation has been done on two months (March and August).

**Table 3:** Specific technics of the panel.

*(A)*	$V_{oc}$*(V)*	$I_{mpp}$*(A)*	$V_{mpp}$*(V)*	P*(W)*
*2.24*	*20.50*	*1.93*	*15.07*	*30*

**Fig. 8:**
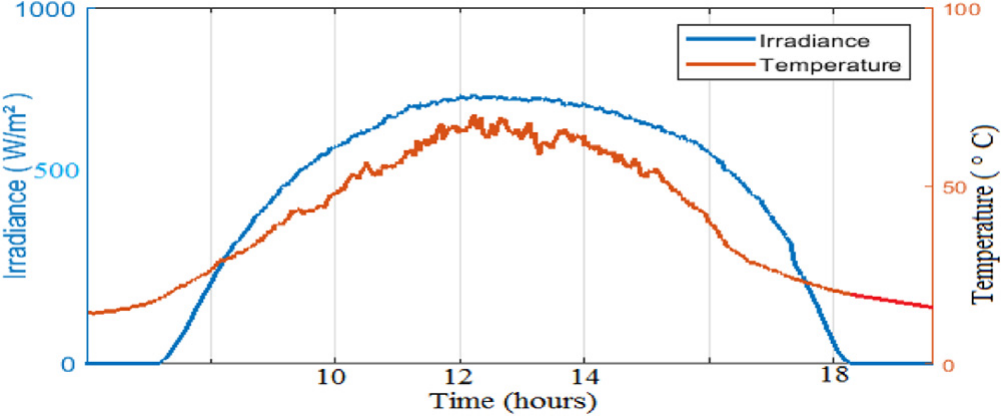
Solar irradiation in March.

Where: P is the electric power of solar panel: $I_{mpp}$, $V_{mpp}$ are the optimal current and the voltage, respectively; $I_{sc}$ is the short-circuit current and $V_{oc}$ is the open circuit voltage. In climatic variations of one day of each month are presented in Fig. 8. March coincides with the dry season where the sky is clear unlike the month of August which coincides with the rainy season.

**Fig. 9:**
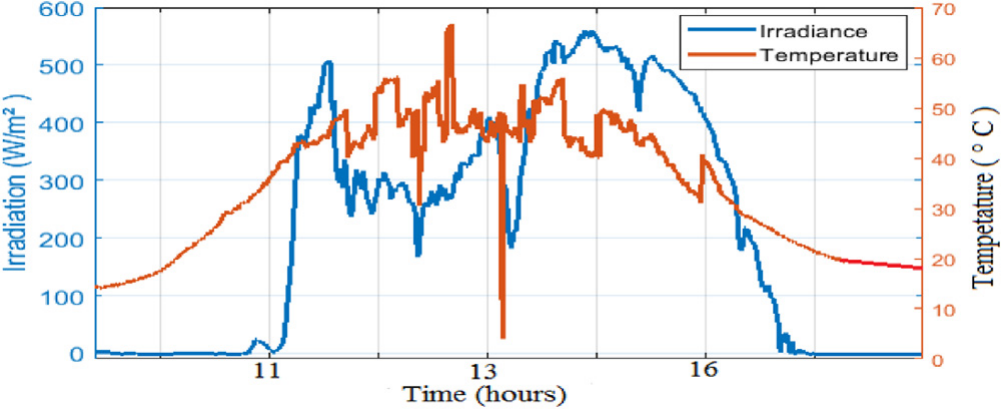
Solar irradiation in August.

**Fig. 10:**
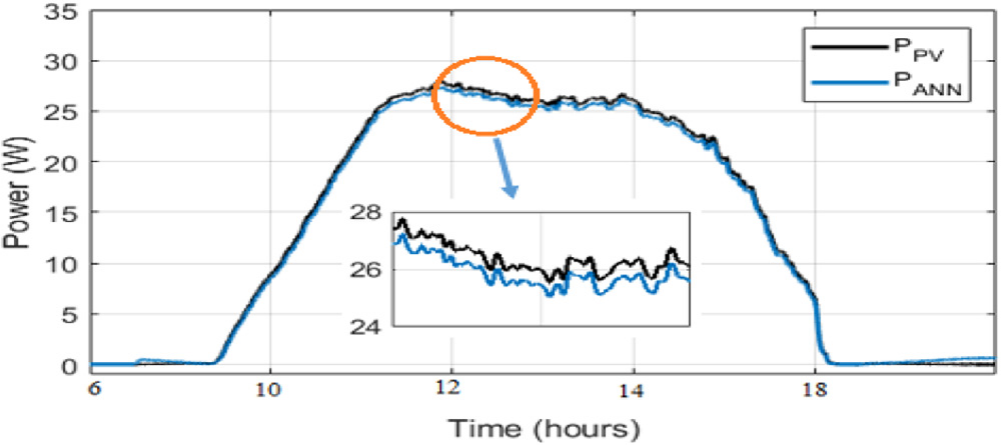
PVs power and ANN MPPT power optimized for a sunny day in March.

**Fig. 11:**
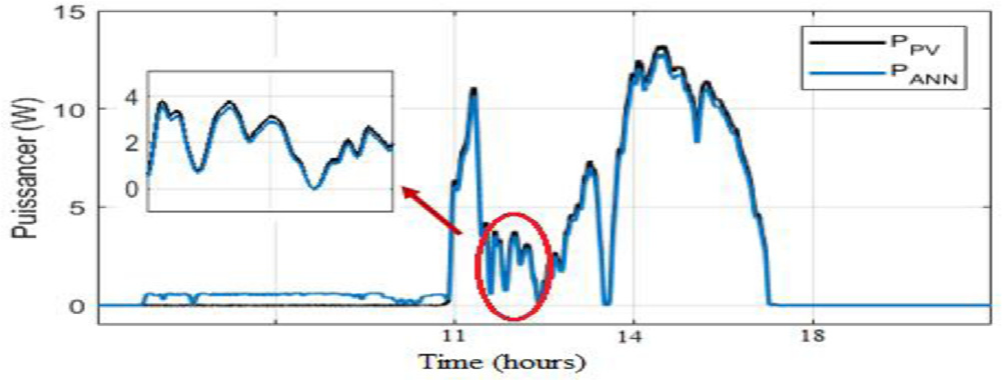
PVs power and ANN MPPT power optimized for a cloudy day in August.

**Fig. 12:**
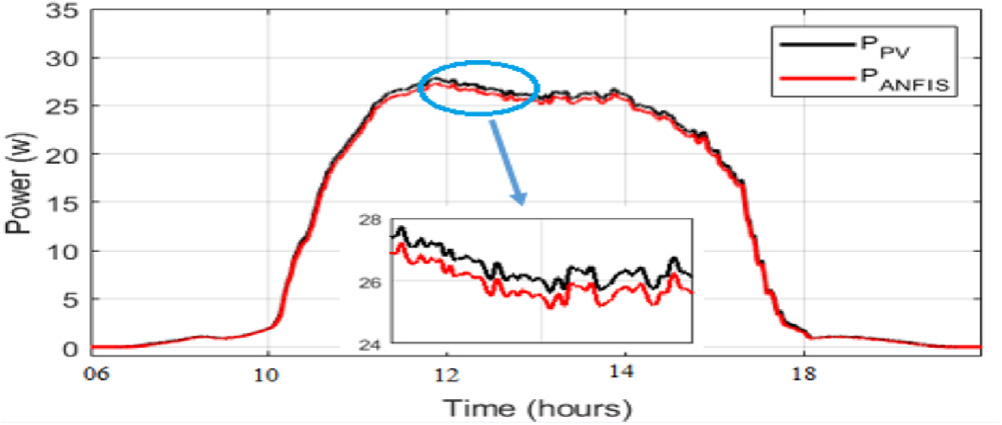
PVs power and ANN MPPT power optimized for a sunny day in March.

**Fig. 13:**
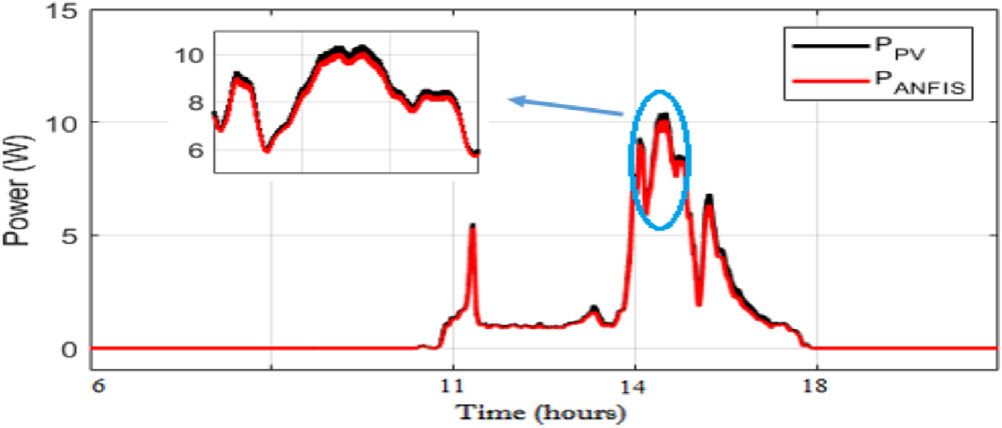
PVs power and ANN MPPT power optimized for a cloudy day in August.

As observed in Fig 8 and 9, the daily weather conditions has not a same repartition, this is characterized by the different profiles of daily weathers (sunny, cloudy). The output power of the PV panel is plotted Fig.10, 11, 12 and 13. As can be seen, both the ANN and the ANFIS methods are identical in all cases of atmospheric variations for each month. In this analysis, we can confirm the two MPPT have a very good solution to track the MPP. This ability to track the MPP for different weather condition is proved from the reached efficiency and a good stability is achieved. Furthermore, the MPPT methods have a high tracking speed capability which allows transferring the PV energy with minimum losses and a test of performance of those controllers is listed in Table 4. And there is a good agreement between the measured and the predicted curves for Month and August. The RMSE, the MAE and the MAPE of an ANFIS are lower than those given with an ANN method for each month.

**Table 4:** Performance evaluators of ANN and ANFIS models.

Month	March			August		
Model	RMSE	MAE	MAPE (%)	RMSE	MAE	MAPE (%)
ANN	0.4472	0.4194	0.0016	0.3868	0.3426	0.0039
ANFIS	0.3569	0.2778	$8.2523\;10^{-4}$	0.1162	0.0508	$4.2429\;10^{-4}$

**Fig. 14:**
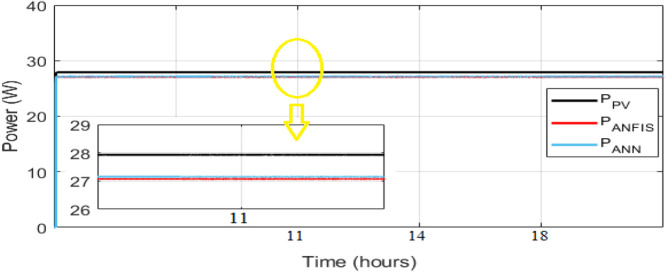
PVs power and ANN and ANFIS powers optimized under STC (1000W/m² and 25°C).

It can be seen from above Table 3 that, in terms of all three error criteria, the proposed ANFIS model obtained better prediction results even in these challenging selected months typified by both weak and strong fluctuation levels. ANFIS model optimizes the prediction accuracy and reduces the two months MAPE to $8.2523\;10^{-4}$ % and $4.2429\;10^{-4}$ % respectively in March and August. ANN model MAPE equal to 0.0016 % and 0.0039 % respectively in March and August. The evaluation criteria in Table 3 clearly demonstrate the accuracy and validity of the ANFIS. A robustness test of the two intelligent controllers is done in order to confirm the most powerful MPPT controller between ANN and ANFIS. This test is performed in standard conditions (STC) (1000W/m² and at 25°C) and the simulation result is showed in Fig. 14.

This result shows that the output of ANFIS and ANN are almost confused with the PV output power. Here we can conclude that ANFIS is most robust than the ANN and Table 5 confirm this robustness. This can be explained ANFIS is more robust because it have the ability to adapt to this conditions and ANN has the advantage of generalization after the learning process.

**Table 5:** Comparison result based on different evaluator factor in Standard conditions (1000W/m² and 25°C).

*STC*	*1000 W/ m² and 25°C*		
*Model*	*RMSE*	*MAE*	*MAPE (%)*
*ANFIS*	*0.8510*	*0.8494*	*0.029*
*ANN*	*0.8523*	*0.8508*	*0.029*

The simulation results is done under Matlab/Simulink and several tests are done to pronunciation on the most powerful MPPT methods. The statistic evaluators found with ANFIS is lower than the ANN for the performance test Table 4. In robustness test ANN show it good results with low errors Table 5 and the ability to adapt for a new variations of meteorological conditions. Without the evaluators tests the simulation time of the ANN is fast compared to the ANFIS method. At the end of this study we show that ANFIS is more powerful than ANN MPPT controller because of it ability of learning and adaptation to the weather condition. The aim of this study is to confirm the performance of the ANFIS compared to the ANN. Many research listed below have done this comparison and at the difference of our study this comparative study is done by using real weather conditions and the simulation is done in two seasons (dry and rainy). The Table 6 summarized a comparative study of the two intelligent methods. This methods have a fast convergence speed in Figs. 1 and 2, we show ANFIS structure is more complicate than ANN because it is a hybrid method combined the advantages of the ANN and FL. ANN is less expensive than ANFIS and it hardware implementation is less complicate.

**Table 6:** Comparison between ANN and ANFIS models.

Model	Convergence speed	Cost	Complexity
ANFIS	Fast	high	very
ANN	Fast	Medium	Less

## Conclusion

A comparative study of two intelligent controllers for optimizing the PVs power in non-uniform weather conditions is done. Several factors are affecting the performance of PV panels, such as partial shading conditions and non-uniform weather conditions. In tropical area, the climatic conditions vary in each seasons and most significant in rainy season due to the clouds. This paper analyses the performance of an ANN and ANFIS MPPT controller of a PVs in non-uniform climatic conditions in two months (March and August). In order to obtain the most significant efficient controller, several mathematical coefficients have been adopted to determine the validity of accurate results such as MAPE, MAE and RMSE. The results show that ANFIS is most powerful than ANN because of its ability to adapt in non-uniform weather condition and it capability to generalize. In future, we would like to study in the whole month of the year and in another area.

## Declaration of Competing Interest

The authors declare that they have no known competing financial interests or personal relationships that could have appeared to influence the work reported in this paper.

## Data Availability

Data will be made available on request.
